# Spotlighting main group elements in polynuclear complexes

**DOI:** 10.1039/d1sc90011k

**Published:** 2021-02-17

**Authors:** François P. Gabbaï, Cameron Jones, Connie C. Lu

**Affiliations:** Department of Chemistry, Texas A&M University College Station Texas 77843-3255 USA francois@tamu.edu; School of Chemistry, Monash University PO Box 23 VIC Australia cameron.jones@monash.edu; Department of Chemistry, University of Minnesota-Twin Cities 207 Pleasant Street SE Minneapolis Minnesota 55455 USA clu@umn.edu

## Abstract

François Gabbaï, Cameron Jones and Connie Lu introduce the *Chemical Science* themed collection on the topic of main group elements in polynuclear complexes.
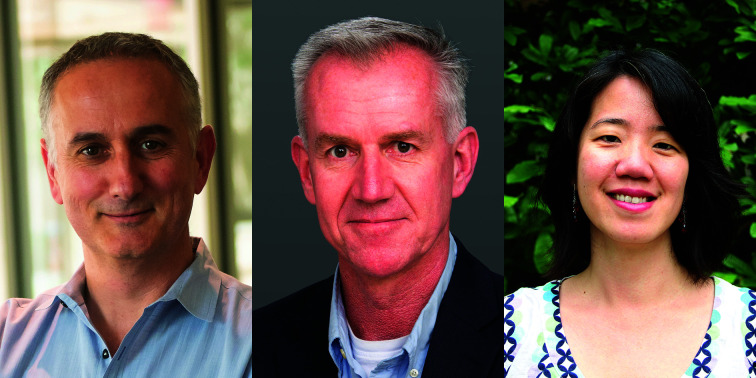

Efforts towards the incorporation of main group elements in polynuclear motifs or in the coordination sphere of transition metals have been a prevalent theme of coordination chemistry, and one that has delivered notable advances in the area of structure and bonding. In the past decade, this field has witnessed an increased emphasis on the influence of the main group moiety over the reactivity or physical properties of the resulting constructs. Through a collection of both invited and selected articles, this themed issue puts the spotlight on this developing field, while at the same time illustrating far-reaching applications in the areas of small molecule activation, catalysis and molecular magnetism.

A number of papers in this themed issue highlight the significant recent progress that has been made in the development of homo- and heterometallic systems incorporating s- and p-block elements, both in low oxidation states (often element–element bonded) and normal oxidation states. These have found particular use as low toxicity, earth abundant alternatives to late transition metal complexes in stoichiometric and catalytic transformations of small molecule substrates to value added products. This theme is introduced in primary articles dealing with the reactivity of magnesium-based systems. As shown by Jones, Maron and co-workers, magnesium(i) dimers (LMg–MgL, L = β-diketiminate) are activated by coordination of simple Lewis bases, and are subsequently able to reductively couple carbon monoxide to form the deltate and transient ethenediolate dianions (C_*n*_O_*n*_^2−^, *n* = 3 and 2, respectively; DOI: 10.1039/D0SC00836B). In another contribution, β-diketiminato-stabilised magnesium diboronates are shown by Hill, McMullin and co-workers to act as rare “masked” sources of nucleophilic boryl anions for the synthetic transformation of imines (DOI: 10.1039/C9SC02087J). These two papers integrate with the content of two reviews that highlight the unique structures and reactivity of polynuclear complexes containing low valent group 2, 13 and 14 elements. One of these reviews, by Inoue and co-workers, focuses on the structures of ditetrelenes (R_2_E^II^=E^II^R_2_, E = group 14 element) and ditetrelynes (RE^I^≡E^I^R), and their remarkable reactivity towards small molecules (DOI: 10.1039/D0SC03192E). Another review by Crimmin and co-workers explores the role that magnesium(i) and aluminium(i) reductants play in C–H bond activation reactions, and the synergy that may arise when the main group reagent is combined with a transition metal (DOI: 10.1039/D0SC03695A). Showcasing the value that s-block metals may display in their normal valence, Williams and coworkers describe macrocyclic Mg^II^/Zn^II^ heterodinuclear complexes as highly effective catalysts for epoxide/CO_2_ ring opening co-polymerization (DOI: 10.1039/C9SC00385A). The broader significance of this concept is developed in a review on heterobimetallic complexes containing s-block metals, in which Hevia and her co-worker highlight the unique ability of such complexes to support cooperative catalysis (DOI: 10.1039/D0SC05116K). The Lewis acidity of s-block cations can also be harnessed to manipulate the covalency of metal–ligand interactions, as elegantly demonstrated by Arnold, Love, Vitova, Schreckenbach and co-workers, who investigate a series of uranyl(v) complexes featuring U=O–E motifs (E = group 1 or 2 element, DOI: 10.1039/C8SC05717F).

Reduced polynuclear main group complexes can also provide new platforms for the discovery of atypical reactivity as illustrated by Kong and co-workers, who report mono-base-stabilized 1,2-diboranylidenehydrazines, a set of compounds that feature an unprecedented BNN-1,3-dipole that readily adds to arenes or small molecules such as CO_2_ (DOI: 10.1039/D0SC02162H). In keeping with the theme of reactive diboron-containing units, Braunschweig and co-workers show in another captivating report that B–B triply-bonded diborynes can add to diboranes to afford B_4_ chains, a transformation that could pave the way to new polymers with polyboron units in the main chain (DOI: 10.1039/C9SC02544H). The synthetic potential offered by low oxidation state main group elements comes to the fore in two additional reports, both dealing with Si_6_ clusters. In the first one, Scheschkewitz and co-workers show that these silicon clusters can be functionalised with tetrylene substituents, and can act as ligands towards group 9 metal fragments, yielding complexes which act as catalysts for alkene isomerisations (DOI: 10.1039/D0SC02861D). A second report by Lips and co-workers describes highly unsaturated and structurally dynamic Si_6_R_4_ species (R = amide) with exposed silicon vertices (DOI: 10.1039/D0SC01427C). Exposed silicon moieties can also be appended to classical ligands as demonstrated by Roesky and co-workers who report on cyclopentadienyl ligands substituted by a silylene (R_2_Si:). These ligands not only act as two-electron Si donors towards transition metal fragments but also undergo isomerization or deprotonation reactions leading to sila-fulvenes (DOI: 10.1039/D0SC04174B). Reduced group 14 elements can also be directly incorporated in the five-membered ring of cyclopentadienyl-like ligands as illustrated by Müller, Albers and co-workers in a contribution dealing with the germacyclopentadienediyl [K_2_(:GeC_4_R_4_)] as an η^5^-ligand and its conversion into the first germaaluminocene, [Cp*Al(η^5^-:GeC_4_R_4_)] (DOI: 10.1039/D0SC00401D).

As stated in the introductory paragraph, positioning main group elements in the coordination sphere of transition metals provides access to unusual reactivities, as in a contribution by Ozerov and co-workers (DOI: 10.1039/D0SC04748A) who demonstrate the reversible addition of ethylene to a boryl-based bis(phosphine) iridium pincer complex. A unique aspect of this contribution is the concomitant participation of the iridium and boron centres in the coordination of the hydrocarbon ligand. The ability of boron to cooperate with an adjacent transition metal centre is again a leading theme in two additional contributions selected for inclusion in this issue. The first one concerns the reversible addition of H_2_ across an Ni–B bond, as elegantly documented by Rodríguez, Lledós and co-workers (DOI: 10.1039/D0SC06014C), who also used a boryl-based pincer as a supporting ligand. Exploiting the somewhat counter-intuitive reality that gold is more electronegative than boron, Yamashita, Lin and co-workers show that gold(i) diarylboryl complexes react as gold-based nucleophiles with organic reagents bearing C=O and C=N bonds (DOI: 10.1039/D0SC05478J). The unique reactivity of late transition metal–boryl linkages pervades in another contribution by Conejero, Lledós and co-workers who detail the highly choreographed addition of boranes such as HBpin and HBcat to a cationic, T-shaped, cyclometallated Pt(ii) bis-carbene complex (DOI: 10.1039/D0SC05522K). Isolated species include σ-BH Pt^II^ complexes, *en route* to the formation of T-shaped Pt^II^ bis-carbene complexes. Last, Tilley, Eisenstein and co-workers remind us of the importance of main group hydrides in catalysis in a contribution that pinpoints the intermediacy of dinuclear nickel–silyl species in an alkene hydrosilylation reaction mediated by a cationic nickel complex (DOI: 10.1039/D0SC00997K).

Within the theme of heterometallic cooperativity, we highlight three articles where group 13 elements were introduced into transition metal complexes to promote small-molecule activation. In each report, a unique ligand design is used to juxtapose the transition metal centre with the group 13 element(s). Szymczak and co-workers appended two Lewis acidic borane groups to a pincer ligand *via* flexible linkers. The pendant boranes were critical for the stabilization of a rare high-spin Fe^II^ dihydride complex by forging Fe–H → B interactions (DOI: 10.1039/C9SC00561G). Upon exposure to an arylisocyanide, a good π-acid, the reductive elimination of H_2_ ensued to form the iron(0) complex. Such a step is reminiscent of the E_4_ intermediate in nitrogenase, which is proposed to release the obligatory H_2_ equivalent upon binding of N_2_ [see *Chem. Rev.*, 2014, **114**, 4041]. Envisioning a more active role for boranes, Harman and co-workers use the diboraanthracene platform, whose redox flexibility and dynamic Lewis acidity can be orchestrated to promote reactivity at the bound transition metal (DOI: 10.1039/C9SC02792K). The authors isolate a key Au borohydride intermediate that reduces CO_2_ to formate, and close a synthetic cycle from CO_2_ to formic acid using only proton and electron equivalents. Moving down the group 13 to the heavier congeners, Lu and co-workers show that the choice of the heavy group 13 ion (Al, Ga, or In) that is directly appended to a nickel(0) centre can significantly tune the Ni electronics (DOI: 10.1039/C9SC02018G). In comparing a triad of non-classical Ni(η^2^-H_2_) adducts, the identity of the group 13 ion was found to perturb the free energy and activation energy of H_2_ binding by ∼5 kcal mol^−1^. Lastly, in a timely review, Takaya details the growing momentum of using main group/metalloid complexes as supporting ligands for transition metal-based catalysis (DOI: 10.1039/D0SC04238B). Takaya’s review presents illustrative examples to showcase the diverse main group elements (groups 13–15) and strategies that are being harnessed for transition metal catalysis.

Moving down the periodic table to the f-elements, several articles explore heterometallic lanthanide and actinide complexes that fundamentally challenge our understanding of bonding and electronic structure. Using mixed arene π-ligands, Liddle and co-workers isolated an unusual bent Th “sandwich” complex that is stitched by K^+^ ions into a tetrathorium cluster (DOI: 10.1039/D0SC02479A). Diaconescu, Huang and co-workers report inverted sandwich complexes of Sm and Y featuring a bridging biphenyl ligand and bridging K^+^ ions (DOI: 10.1039/D0SC03555F). Depending on the lanthanide element, these inverted sandwiches feature Sm^III^–arene–Sm^III^ or Yb^II^–arene–K^+^ bonding interactions, where the biphenyl ligand is formally tetraanionic or dianionic, respectively. Freedman and co-workers conducted an in-depth study on the electronic structures of Sn-based heterometallics that contain a direct bond between Sn and a first-row transition metal that is varied from Mn to Ni (DOI: 10.1039/D0SC03777J). The authors make a striking comparison between the high-spin configurations of the 3d ions and those of typical Ln coordination complexes, wherein the coordinate bonds are more ionic. They rationalize that the Sn group behaves as an inverted, weak-field ligand due to the large energy mismatch between the Sn 5s/5p and 3d atomic orbitals [see *Chem. Rev.*, 2016, **116**, 8173]. Controlling spin states is only one of several requisites for the design of single molecule magnets (SMMs). Layfield, Mansikkamäki and co-workers report a triad of dinuclear dysprosium complexes, where the exogenous borohydride donor is varied in both number and coordination (terminal to bridging) (DOI: 10.1039/D0SC02033H). The authors observed a favourable increase in the effective energy barrier for a dinuclear dysprosium complex with a Dy : BH_4_ ratio of 2 : 1. Lastly, Nippe, Chibotaru and co-workers explore magneto-structural relationships in a series of trigonal prismatic Ln^III^ complexes (Gd to Lu) that are scaffolded by three doubly deprotonated ferrocene (FeCp_2_)^2−^ ligands and capped by Li^+^ ions (DOI: 10.1039/D0SC01197E). By virtue of its size and axis of anisotropy, the authors were able to engender SMM behaviour for the Ho^III^ complex. The authors demonstrate that the Ln size and the nature of the Li^+^ solvate both influence the twist angle, where the ideal trigonal prism geometry (twist angle of 0°) results in the large anisotropy that is conducive to SMM behaviour.

To illustrate the diversity of the field, this themed issue also highlights several additional contributions dealing with atypical phosphorus-containing ligands. For example, Scheer and co-workers show that the four-membered cyclo-P_4_ ligand of organometallic tantalum complexes can be used as a square building block for the construction of molecular capsules upon combination with silver cations and an appropriate template (DOI: 10.1039/D0SC03437A). Two additional contributions document recent trends at the confluence of traditional organophosphorus chemistry and coordination chemistry. Gessner and co-workers review the unique properties of phosphorus ylides and their ability to stabilize low-valent main group species, leading to the formation of new main group ligands for transition metal-based catalysis (DOI: 10.1039/D0SC03278F). The second contribution comes from Normand, Sosa Carrizo and co-workers who decipher the ambiphilic properties of bis(iminophosphoranyl)phosphide ligands and suggest that they be regarded as containing a triphosphenium coordinating unit (DOI: 10.1039/D0SC04736H).

This themed issue was assembled with the intent of spotlighting the role played by main group elements in polynuclear complexes. We hope that those reading these articles will appreciate the topical diversity of this research field, its relevance to various areas of chemistry, and the numerous future research opportunities it presents.

## Supplementary Material

